# Female naturalists and the patterns of suppression of women scientists in history: the example of Maria Sibylla Merian and her contributions about useful plants

**DOI:** 10.1186/s13002-023-00589-1

**Published:** 2023-05-12

**Authors:** Fernanda Mariath, Leopoldo C. Baratto

**Affiliations:** Laboratory of Applied Pharmacognosy, Faculty of Pharmacy, Universidade Federal do Rio de Janeiro, Rio de Janeiro, Brazil

**Keywords:** Natural history, Historical ethnobotany, Naturalists, Women in science, Surinam, South America, Biodiversity, Useful plants

## Abstract

**Background:**

This work reunites many women naturalists who registered knowledge about native flora in scientific expeditions around the globe between the seventeenth and nineteenth centuries. Since male naturalists are more recognized in this period of time, we aimed to list female naturalists that published plant descriptions and observations, focusing on the work of Maria Sibylla Merian and to analyze her trajectory as an example to discuss the patterns of the suppression of women scientists. A second aim was to inventory the useful plants described in Maria Sibylla’s *Metamorphosis Insectorum Surinamensium* and find pharmacological evidence about the traditional uses described for those plants cited as medicinal and toxic.

**Methods:**

A survey of female naturalists was carried out by searching information in Pubmed, Scielo, Google Scholar and Virtual Health Library. Once Maria Sibylla published her book *Metamorphosis Insectorum Surinamensium* by her own, without male co-authors, and also this book is one of the only to have text and illustrations altogether and there are reports indicating information on useful plants in this work, she and her book were chosen as subject of this research. All the information was tabulated by dividing the plants into food, medicinal, toxic, aromatic or other uses. Finally, with the combinations of the scientific name of medicinal and toxic plants with information about their popular uses, a search was carried out in databases in order to indicate current pharmacological studies that reported evidences about the traditional uses described.

**Results:**

We found 28 women naturalists who participated in scientific expeditions or trips, or in a curiosity cabinet, or who were collectors of Natural History between the seventeenth and nineteenth centuries. All these women illustrated botanical species and/or recorded their everyday or medicinal use or reported their observations in the form of a published work, letters or diaries. Also, the trajectory of Maria Sibylla Merian revealed that her scientific relevance has been neglected from the eighteenth century by mechanisms of suppression, most of the time by male depreciation, which can be seen as a pattern for suppression of women in science. However, Maria Sibyllas’ contributions have been valued again in the twenty-first century. In Maria Sibylla’s work, 54 plants were identified, 26 of them used for food, 4 of them aromatic, 8 medicinal, 4 toxic and 9 other uses.

**Conclusion:**

This study evidences that there are female naturalists whose work could be an important source for ethnopharmacological studies. Researching about women scientists, talking about them and highlighting the gender bias present in the scientific academy about the way the history of science is told is essential for the construction of a more diverse and richer scientific academy. The traditional use of 7 of 8 medicinal plants and 3 of 4 toxic plants reported was correlated with pharmacological studies, highlighting the importance of this historical record and its potential to direct strategic research in traditional medicine.

## Background

Historical ethnobotany is a valuable tool that investigates the relationships between people and plants within the context of historical, ecological, social and traditional aspects, with one of its aims being the search for new useful plants [1]. Thus, researching historical records (oral, written and/or iconographic sources of information) is very important in order to rediscover the ethnopharmacological value of plant uses. Primary written sources are classified as official documents (e.g., laws, decrees, offices of governments and reigns), manuscripts (travel journals of naturalists and travelers, codes, religious books etc.), letters, and medical prescriptions [2].

Much of the historical information about the use of plants native to the Americas, for example, was compiled by naturalists who traveled or lived on the continent between the sixteenth and nineteenth centuries [3]. One of the first mentions of the Latin American flora and its uses dates back to 1520, when the Italian scholar Antonio Pigafetta referred to the medicinal and useful plants of southern Patagonia aboard Ferdinand Magellan’s expedition [4]. The first inventories of Mexico were compiled from works by the Spanish naturalists Francisco Hernández (1570–1577) with translation from Latin to Spanish by Francisco Ximenez de Quesada (1615) [5] and Nicolas Monardes (1574) [6]. Information regarding useful plants in the Guianas and South America in the seventeenth century is scarce, with Maria Sybilla Merian’s [7], Willem Piso’s and George Marcgraf’s [8] works being the oldest records. The German naturalist Marcgraf and the Dutch physician Piso explored northeastern Brazil [8]. Paul Hermann, a German botanist at Leiden University, owned the oldest documented herbarium collection of Surinam and the Guianas region [9, 10]. The Irishman Hans Sloane cataloged Jamaican plants between 1687 and 1689 [11].

The eighteenth and nineteenth centuries were a period of major expeditions worldwide [12]. Important Spanish expeditions were led by Hipólito Ruiz and Antonio Pavón (1777–1786), José Celestino Mutis (1782–1808), and Martín Sessé (1788–1796), who stimulated the description of regional floras, e.g., Flora Mexicana (1885) [13], *Plantae Novae Hispaniae* (1889–1893) [14], *Flora Peruviana et Chilensis* (1798–1802) [15], and Flora de la Real Expedición Botánica del Nuevo Reino de Granada (1828) [16]. In addition, French naturalists reported Latin American natural resources described in expeditions conducted by Charles Plumier (1689–1697) to the West Indies (Martinique and Haiti) [17], by Joseph de Jussieu (1735–1771) to Peru [18], by Charles-Marie de la Condamine (1735–1744) from the Peruvian Andes to the Atlantic Ocean through the Amazon river [19], and by Jean-Baptiste C. F. Aublet (1762) in the French Guiana [20], among others [21]. Some of the most important naturalists of that time were Alexander von Humboldt and Aimé Bonpland who explored South and Central America between 1799 and 1804, collecting more than 6000 plant specimens [22].

Although the findings and observations of all these naturalists had been published at their times, it is very common to note that the ethnobotanical data in their works still did not receive necessary attention. The discussion of this information recorded by naturalists is essential, even more so in the scenario of accelerated deforestation of South American native ecosystems, which contributes to the loss of knowledge about medicinal plants used in traditional medicine [23]. In addition to the records obtained from a primary source (either the people who used these plants or those who promoted their use), the information dates back to a time when native vegetation was practically intact and traditional medicine was based on native plants [24], in contrast to the current reality marked by intense deforestation and the introduction of several exotic plants in traditional medicines.

### Neglecting female naturalists in science

A survey among key natural history anthologies for presence of chapters on/by women scientists found a generally absence of women, reporting that natural history compilations shows far more entries from and about men [25]. Women’s trajectories are usually neglected by the History of Science. In numerous fields, exploring this history brought many women who have been neglected [26]. It is notable that all tables and citations of travelers and naturalists mostly or exclusively refer to male names [23, 27–29]. Looking with more attention, women are cited in footnotes or superficially in texts about scientific expeditions as wives and daughters of male leading naturalists. There are a lot of possible reasons that could prevent women from becoming a naturalist, like being unable to join an academic society at the time or not being encouraged to pursue a research path or not receiving credit for their work [25]. Schiebinger [30] also reports that an important obstacle was the fact that women were not hired by commercial companies, scientific academies or governments, which were the main funders of these trips. During centuries men were considered to be rational and objective like science should be, while women were seen as emotional and subjective. Exploring expeditions and field work were associated with masculinity and heroism, once the conditions of the trips and the new places with different kinds of people were considered to be dangerous and inappropriate to a woman [31].

### Crystal labyrinth

In the twenty-first century, women still face obstacles along their academic path. These barriers are described by the Brazilian researcher Betina Lima as the crystal labyrinth: the transparency of the crystal represents the absence of legal devices that prevent the performance of female professionals, and the labyrinth is due to these invisible barriers being present throughout the entire career of women [32].

Two main forms of exclusion of women in science can be observed: horizontal and vertical exclusion [32]. The first is related to the choice of an area, with the absence of women from some specific areas of knowledge. An example is that women are a majority in certain areas of higher education in Brazil, representing more than 70% of graduates in health, education and social sciences courses and more than 50% in the other major areas of undergraduate courses, whereas they represent a little more than 30% and 10% of graduates in engineering and informatics respectively. That is, even though female access to university education has increased, in some areas there is still little female representation [33].

The second refers to the permanence and rise of women in the scientific academy, with few women in prestigious and leadership positions. A representation of vertical exclusion is the election of the researcher Helena Nader as the first female president of the Brazilian Academy of Sciences (ABC) only in 2022, 105 years since the foundation of this agency. The traditional Royal Society of the UK, for example, was never chaired by a woman and was founded in 1660. The Academies of Germany and Italy also never had female presidents [34]. The French Academy of Sciences, created in 1699, had only one woman among its 303 presidents, the biochemist Marianne Grunberg-Manago [35]. The National Academy of Sciences of the United States, founded in 1863, currently has its first female president, Marcia McNutt, a geophysicist who was editor-in-chief of the scientific journal Science [36]. And the Nobel Prize, one of the most prestigious prizes in science, has been awarded thus far to only 6.36% female scientists [37].

### Matilda’s effect

Many people may think that, due to the historical context and the social impositions on the female sex, no women have been cited as naturalists because they have not adventured on scientific expeditions around the world [38]. However, there is a systematic devaluation of women in the history of science and a way of exclusion. Regardless of their area and of how much they have risen professionally in their time, it is very rare to find women scientists being cited as relevant examples for science.

With the rescue of women scientists neglected in the history of science, how famous should these women essentially be? How specific and how widespread should a scientific reputation be? If it were possible to create a scale or measure, could it be determined how absurd it is for a scientist to be ignored or forgotten? After all, not everyone can or should be remembered by everyone. But if it were just a matter of reflecting merit, similar achievements should receive a similar reputation or recognition. And once the reputation is achieved, it should persist for generations to come. This rarely happens in the case of women in science: not only do those who were not recognized in their own time remain so, but others who achieved prestige in their time are neglected from history [39].

Should not the first female president of the French Academy of Sciences be remembered? Wouldn't the fact that the same Academy, which rejected the celebrated Marie Sklodowska Curie for membership, elected a woman as president in 1995 be a significant enough milestone for Marianne Grunberg–Manago's name to enter the scientists' hall of fame? Or the fact that she discovered the first enzyme that synthesizes nucleic acid? And how about the female scientists who were awarded the Nobel Prize? Would not being recognized by one of the most prestigious awards prove the relevance of their contributions? Donna Strickland herself, the 2018 Physics laureate, admitted not knowing the only other female Physics previously laureate (besides Marie Curie), Maria Goeppert-Mayer; Andrea Ghez is currently the fourth laureate in that category. Does not a Nobel Prize guarantee recognition in your own area of expertise?

The Matilda’s Effect was first described by the nineteenth century abolitionist suffragette Matilda Joslyn Gage (1826–1898) in her essay Woman as Inventor and was coined by science historian Margaret Rossiter in 1993 [39, 40]. This effect results in an erasure of women scientists; their contributions to science are not given due credit or, when they are, the names of these women are nonetheless neglected from history.

Then, the question arises whether it is recognized that women carried out scientific expeditions from the seventeenth to the nineteenth century, mainly in South America, documenting the use of plants, especially medicinal ones, and how much of the valuable information collected by them is being lost or little explored.

### The case of Maria Sibylla Merian

It is possible to contribute to the reversal of scarcity of female names and deconstruct the image that sciences were an exclusively male practice in the past by highlighting experiences of women and recognizing their participation in the production of knowledge [31].

**Fig. 1:**
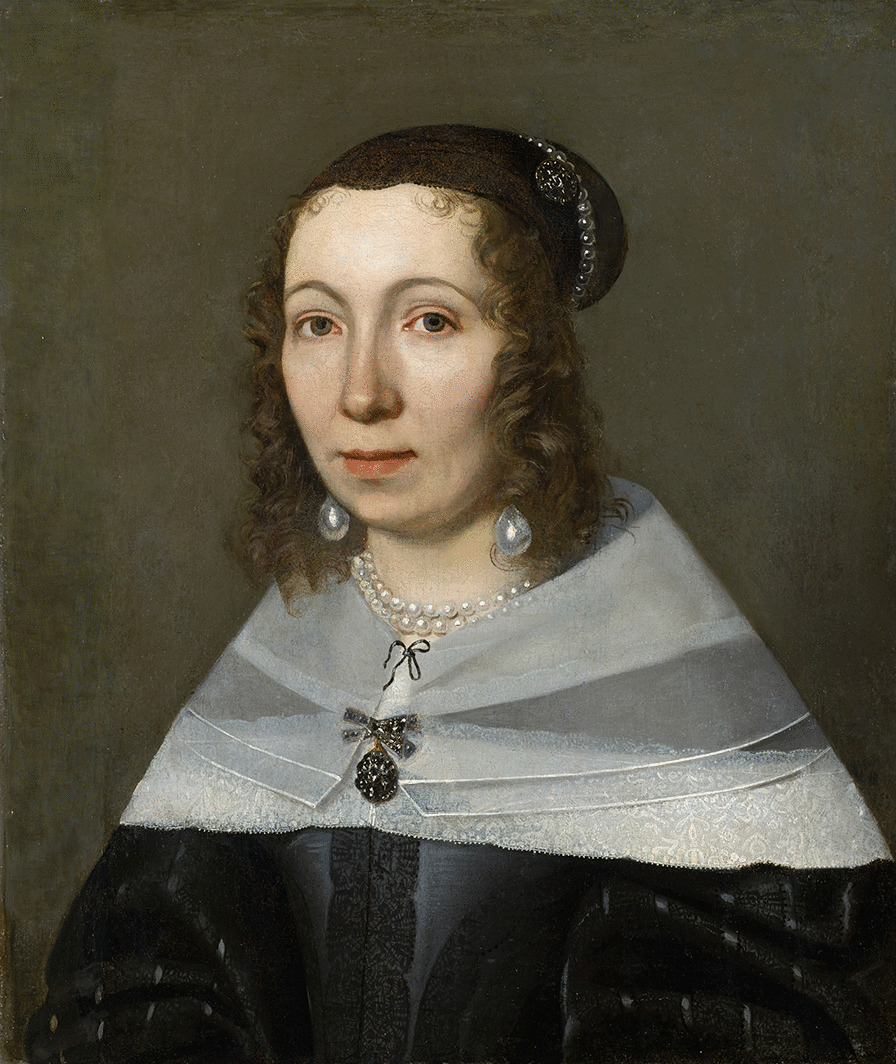
Portrait of Maria Sibylla Merian (1647–1717) painted by Jacob Marrel in 1679

One name that stands out is Maria Sibylla Merian (1647–1717) (Fig. 1), a German researcher who published a book on insects and plants in Surinam after a self-funded scientific expedition [41]. Merian traveled on her own in pursuit of her own scientific questions and that is why her trajectory differs from the majority of other women naturalists who were normally in the position of colonial wives, accompanying the project of their husbands [30].

From childhood, she studied insects, mainly butterflies and moths, and also collected caterpillars in gardens. For years, she systematically observed and noted the behavior of caterpillars, drawing each stage of their development [42, 43]. In 1679, she published the first edition of “*Der Raupen Wunderbare Verwandelung, und sonderbare Blumennahrung*” [44] (*The Wonderful Transformation of Caterpillars and Their Eating Habits*), containing 50 plates of engravings and commentaries on European insects; later she would publish two more editions of this book [45].

**Fig. 2:**
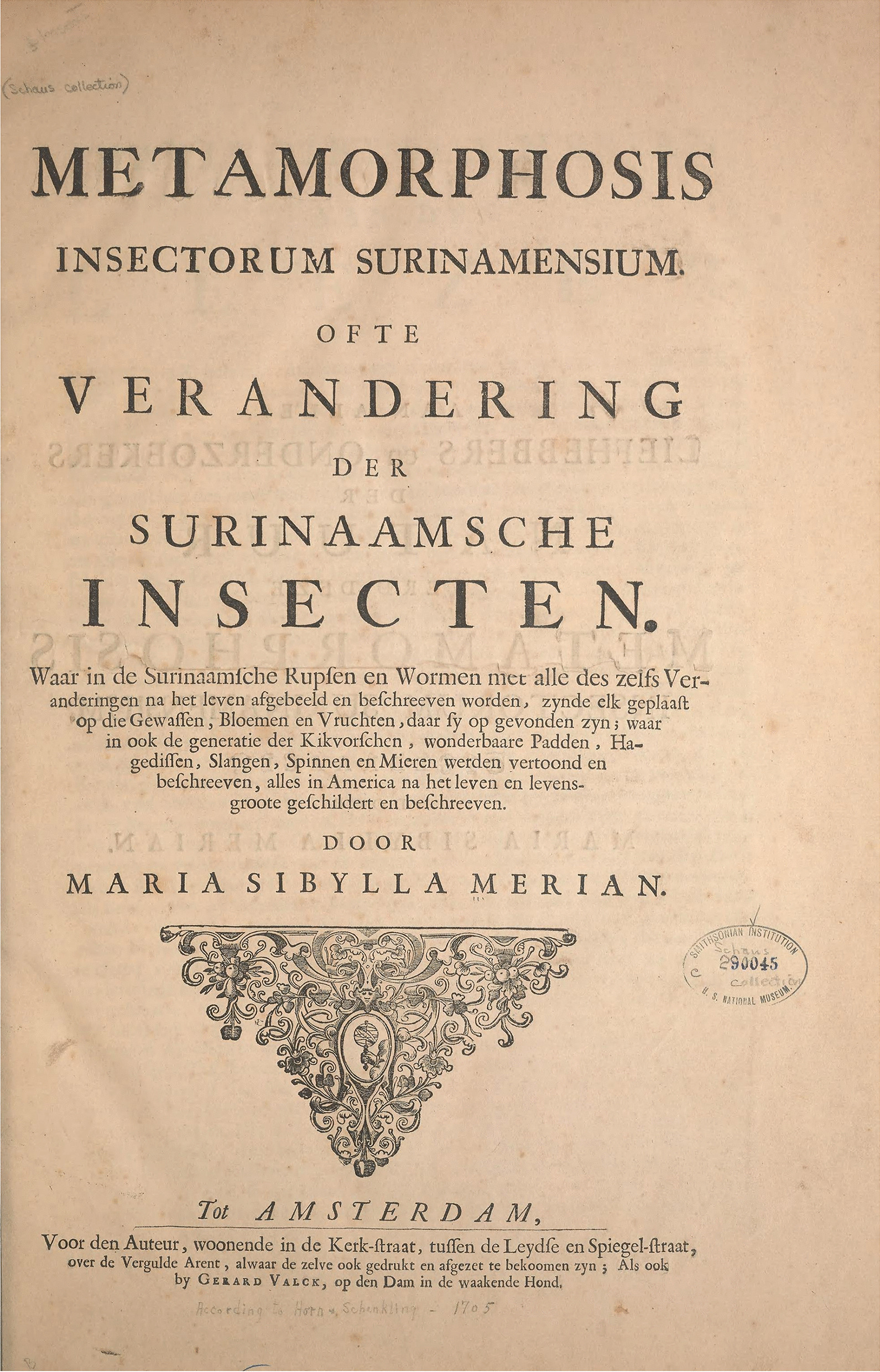
Cover page from the Dutch version of Maria Sibylla Merian’s *Metamorphosis Insectorum Surinamensium* [7], published in 1705

At age 52, in 1699, Maria Sibylla Merian embarked on a self-funded scientific expedition to Surinam to study insects and plants, without any official sponsorship or a team [30]. Taking her daughter, Dorothea, as a helper, she left with only the money she could scrape together by selling illustrations. Mother and daughter spent two years in Surinam and then returned to the Netherlands, where Merian devoted four years to writing *Metamorphosis Insectorum Surinamensium*, which was published in its first edition in Dutch and Latin in 1705 (Fig. 2) [7]. She collected several specimens, mainly seeds and leaves; however, her voucher specimens did not survive over the years [46].

The aim of the present report is to reunite the name of many women naturalists who registered knowledge about native flora in scientific expeditions around the globe, mainly South America, focusing on the work of Maria Sibylla Merian and discussing the patterns of the suppression of women scientists. Furthermore, we aimed to inventory useful plants described in Maria Sibylla’s *Metamorphosis Insectorum Surinamensium* and find pharmacological evidence about the traditional uses described for those plants cited as medicinal and toxic.

## Methods

The study consists of two parts: first, we conducted a survey of women naturalists with a discussion of patterns in their suppression in the history of science, taking the trajectory of Maria Sibylla Merian as an example. In the second part, we analyzed Merian's contributions regarding the useful plants described in *Metamorphosis Insectorum Surinamensium* and correlated them with current scientific evidence.

Part I: The survey of women naturalists was carried out by searching Pubmed (https://pubmed.ncbi.nlm.nih.gov/), Scielo (https://www.scielo.br/), Biblioteca Virtual em Saúde (https://bvsms.saude.gov.br/) and Google Scholar (https://scholar.google.com.br/), using the following keywords: “women” and “naturalist” or “women travelers”. This survey was carried out in English, Portuguese and French. In addition to the articles and periodicals deposited in the listed databases, books from BiblioMaison (at the French Consulate in Rio de Janeiro) (https://bibliomaison.net.br/) with the same descriptors were consulted. Each woman naturalist’s name found became a descriptor, which was searched separately in Google Scholar. Women who participated in any scientific expedition or trip, or in a curiosity cabinet, or who were collectors of Natural History between the seventeenth and nineteenth centuries, were included. Another criterion for inclusion was having illustrated a botanical species and/or recorded some use of useful plants or reported observations about plants in the form of a published work, letters or diaries.

Part II: The original book *Metamorphosis Insectorum Surinamensium* [7] was consulted in the online catalog of the Biodiversity Heritage Library^1^ using the English transcription of the *Dissertatio de Generatione et Metamorphosibus Insectorum Surinamensium* [47], published by Joannes Oosterwyk in 1719 in Latin, hand-colored, belonging to the John Carter Brown Library. A recent facsimile edition of this book edited by Marieke van Delft and Hans Mulder (2016) was also consulted to compare information [48]. All information was systematized in a table organized by botanical description, illustration number, vernacular name, plant part, origin, traditional uses and observations. Information on toxic and medicinal plants was checked in an official translation into French, *Histoire Générale des Insectes de Surinam et de toute l'Europe* [49], third edition, published in 1711 by M. Buch'oz, with comments and additions from other works published by Maria Sibylla Merian, available in the online catalog of the Bibliothèque Nationale de France. After reading the book, an inventory of the plants was carried out, with notes on descriptions, uses and popular names. These plants were divided into food, medicinal, toxic or aromatic plants (which have some description about good aroma) and other uses (which have some described use that does not fit the other categories, such as wood for construction, painting, and utensils, among others). Subsequently, the identification of the plants was based on the list of species published by the botanists Tinde van Andel, Hajo Gernaat, Auke Hielkema and Paul Maas [48]. Additionally, the updated botanical names were verified in Plants of The World Online (https://powo.science.kew.org/). Finally, using the scientific name of medicinal and toxic plants in combination with their popular use, a search was carried out in Pubmed, ScienceDirect and Google Scholar in order to indicate current pharmacological studies that provide evidence about the described traditional uses.

## Results and discussion

### Women naturalists

Survey in databases allowed us to find 71 women naturalists who lived between the seventeenth and nineteenth century. Of these, 43 were excluded because they did not fit the inclusion criteria. Thus, 28 women naturalists who participated in scientific expeditions or trips, or in a curiosity cabinet, or who were collectors of Natural History during this period of time were included in the present study (Table 1). All of these 28 women illustrated a botanical species and/or recorded some use of useful plants or reported observations about plants in the form of a published work, letters or diaries.

**Table 1:** Women naturalists who participated in scientific expeditions from the seventeenth to the nineteenth century, recording or illustrating useful plants

Naturalist	Nationality	Contributions	References
Adela Breton (1849–1923)	England	Archeologist, explorer and artist She registered an archeological site in Mexico, including observations on landscape and vegetation. She painted many watercolors of plants native to Central America, mainly Mexico, and also authored several papers, discussing her own insights into Pre-Columbian cultures through their artifacts. Her work is recognized as being of great importance for Mesoamerican studies	[50]
Adèle Toussaint-Samson (1826–1911)	France	Author and poet She came to Brazil in 1849 and published “*Une parisienne au Brésil*”, with observations on habits and landscapes	[51]
Albertine Adrienne Necker (1766–1841)	Switzerland	Botanist In collaboration with her husband, professor and botanist Jacques Necker, she published an essay on botanical studies of wild plants, focusing on flowering and fructification	[52]
Anna Blackburne (1726–1793)	England	Botanist and ornithologist She did not participate in scientific expeditions but was a collector of natural history. She corresponded with Linnaeus and other naturalists. She sent Linnaeus specimens of birds and insects that were not described in his *Systema Naturae*	[53, 54]
Anna Jabonowska (1728–1800)	Poland	Collector of natural history She curated a very important European cabinet of natural curiosities which was considered to be one of the most important natural history collections in Europe in the eighteenth century. Several naturalists worked on Jabonowska’s collection and on Albertus Seba’s collection which was part of her cabinet	[55]
Baroness E. de Langsdorff (1812–1889)	France	Traveler and author She came to Brazil in 1842 with the aim of dealing with the marriage of Prince of Joinville and Princess Francisca, D. Pedro I’s sister. Langsdorff published her observations on landscapes in a journal	[56]
Carmen Oliver de Gelabert (not found)	Spain	Traveler and author She came to Brazil in 1870 and published the book “*Viaje poético a Petrópolis*”*,* reporting her visit and impressions about the society and landscapes of the Imperial City Petrópolis, Rio de Janeiro State	[57, 58]
Charlotte Canning (1817–1861)	England	Illustrator and botanist From 1858 to 1861, she traveled to India on several occasions alongside her husband. She collected several botanical specimens and produced more than 300 plant watercolors	[59, 60]
Dorothea Maria Graff (1678–1743)	Germany	Naturalist and illustrator She traveled to Surinam as an assistant to her mother, Maria Sibylla Merian. She became professor of Arts in the Saint Petersburg Academy of Arts and acted as a counselor of arts acquisition for Czar Peter. Also, she became an administrator of Czar Peter’s natural history collection	[45, 61–64]
Elizabeth Cabot Cary Agassiz (1822–1907)	United States of America	Naturalist and explorer She participated in a scientific expedition alongside her husband, Louis Agassiz, from 1865 to 1866; she published her observations about Brazil. She was the author and illustrator of several natural history texts in co-authorship with her husband and godson, including *“A First Lesson in Natural History”* (1859), “*Seaside Studies in Natural History”* (1865), *Geological Sketches* (1867), *A Journey in Brazil* (1868), *Louis Agassiz: His Life and Correspondence* (1885). Agassiz organized and participated in the Thayer expedition to Brazil and the Hassler Strait of Magellan expedition, in southern South America, with her husband	[65–70]
Geneviève de Nanguis-Regnault (eighteenth century)	France	Botanist She was married to the botanist Nicolas François Regnault. She was co-author of *La botanique mise à la portée de tout le monde*, with botanical illustrations containing botanical, historical and medicinal studies	[71]
Ida Pfeiffer (1795–1858)	Austria	Naturalist, traveler and author She traveled around the world for 15 years, coming to Brazil in 1846. She carried a letter of recommendation from Humboldt and other naturalists, who helped with her expeditions. She published many books that were translated into several languages. She became an honorary member of the Society of Geography of Paris and Berlin and also of the Society of Zoology of Berlin and Amsterdam	[72, 73]
Isabel Lady Burton (1831–1896)	England	Traveler and author She traveled to several parts of the world, coming to Brazil in 1865. She published her observations on landscapes and plants	[74, 75]
Jeanne Barret (1740–1897)	France	Naturalist, botanist and explorer She traveled disguised as a man, being the first woman ever to travel around the world in a scientific expedition; she acted as a botanist assistant to Philibert Commerson	[76–78]
Johana Graff (not found)	Germany	Botany illustrator The daughter of Maria Sibylla Merian, she collaborated with the illustrations and publications of her mother and published her own book about Surinam insects	[63, 64]
Madame Roland (1754–1793)	France	Naturalist and apothecary	[52]
Madeleine Françoise Basseporte (1701–1780)	France	Naturalist and painter A botany illustrator with scientific precision, she was invited to contribute to the watercolor collection of the Museum of Natural History of France	[52, 79]
Maria Graham (1785–1842)	England	Naturalist and author Graham traveled to India, Italy and some South American countries such as Chile and Brazil. Her first travel book contained stories about Italy and South America. She was in Brazil between 1821 and 1825, publishing her observations on everyday life and natural resources. She illustrated more than 250 specimens of Brazilian flora and collected plants that were included in the *Flora Brasiliensis* of von Martius	[80, 81]
Marianne North (1830–1890)	England	Naturalist and illustrator She participated in several scientific expeditions aimed at describing plants in their natural environment, illustrating 727 plant genera; she came to Brazil between 1872 and 1873. Some plants were totally unknown and were named after her, such as *Northia seychellana*, *Nepenthes northiana* and *Crinum northianum*; the last one was described based on her drawings. North’s work aroused the interest of several scientists and naturalists, including Charles Darwin	[82–85]
Marie Barbe van Langendonck (1798–not found)	Belgium	Traveler and author She came to Brazil in 1860 and published her travel reports about native vegetation. Her work reported on deforestation and corn and bean plantations	[86, 87]
Marie Le Masson Le Golft (1749–1826)	France	Naturalist focusing on marine biology She published many books including *Balance de la nature* (1784), with notes on hundreds of animals, plants and minerals; *Coup d'Oeil Sur l'État Ancien et Présent du Havre* (1778), with notes on the fauna and flora of a French harbor. She was member of many Science Academies	[88–90]
Marie Robinson Wright (1866–1914)	United States of America	Travel writer She traveled to South America, coming to Brazil in 1889, and published many books reporting observations on landscapes and society. She was the first woman to publish a paper about Iguazu Waterfalls in The National Geographic magazine	[91]
Marie-Armande-Jeanne Gacon-Dufour (1753–1835)	France	Naturalist and agronomist She fought for women's rights to education	[52]
Ottile Coudreau (1870–1910)	France	Cartographer, illustrator and topographer She came to Brazil alongside her husband, Henri Coudreau for research in the Amazon region; after her husband’s death, she took charge of the expedition and published the results on her own	[92–96]
Rose de Saulces Freycinet (1794–not found)	France	Naturalist and travel writer She participated in scientific expeditions and came to Brazil between 1897 and 1820. Her letters with travel observations were published in a diary	[97, 98]
Sarah Bowdich (1791–1853)	England	Botanist, illustrator and taxidermist She followed her husband, the naturalist Thomas Edward Bowdich, in expeditions to Serra Leoa and Gambia; published and illustrated many books	[99]
Teresa von Bayern, Princess of Bayern (1850–1925)	Germany	Naturalist She carried out many scientific expeditions to different continents. She collaborated by collecting specimens for *Flora Brasiliensis* and published the book *Meine Reise in den brasilianischen Tropen* about her Brazilian expedition; she was a member and correspondent of many European scientific institutions. First woman to receive the title *Doctor Philosophiae Honoris Causa*	[100]

**Fig. 3:**
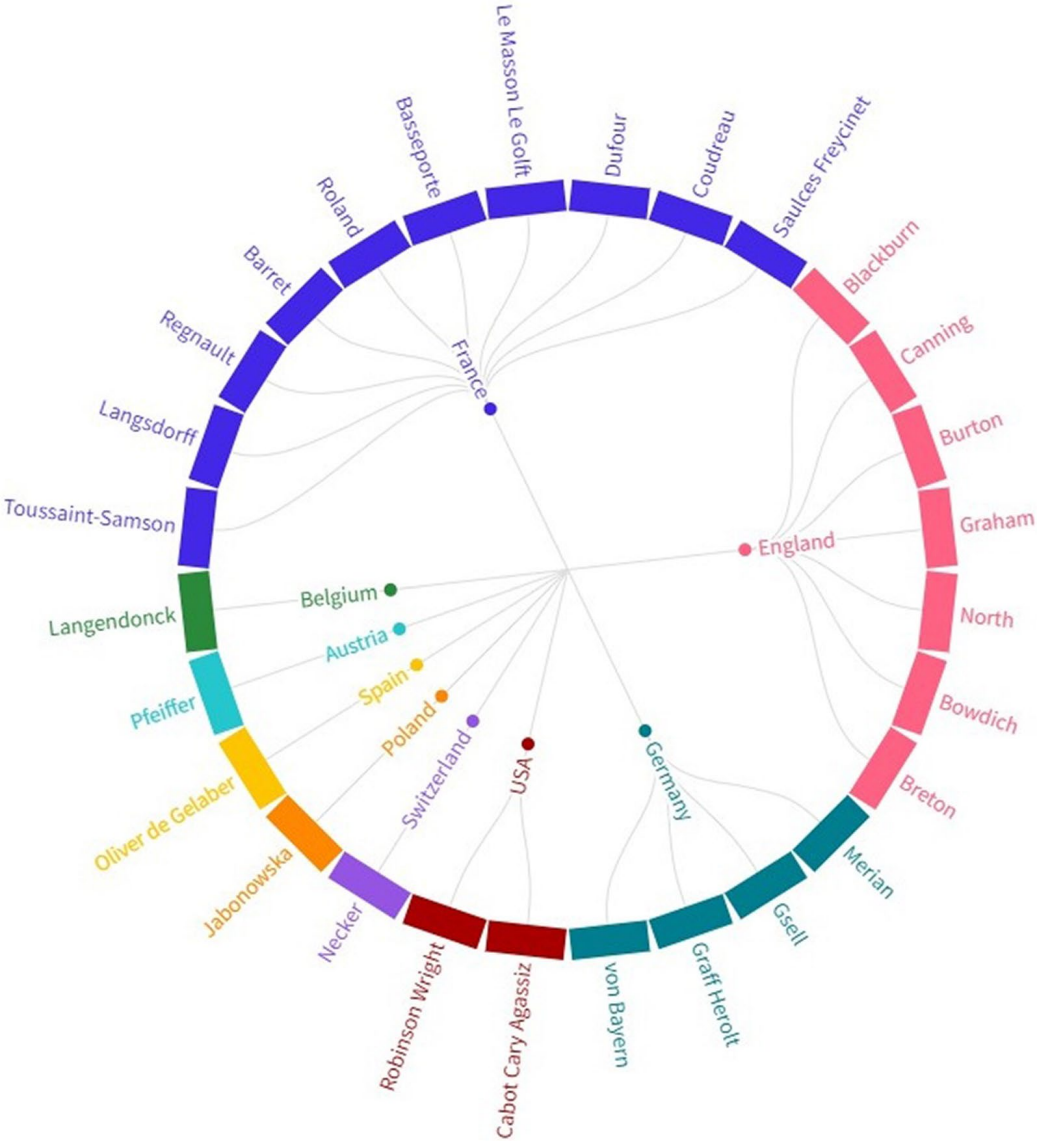
Nationalities of the 28 female naturalists found after a survey in databases

As shown in Fig. 3, the nationality of the majority of the women naturalists included in this work was from an European country: 10 of these 28 women were from France (35%), 7 from England (25%), 4 from Germany (14%), 2 from the USA (7%) and 1 for each of these countries: Austria, Belgium, Spain, Poland and Switzerland. 71% of these women traveled to other countries and 60% of them came to South America; other destinations were India, Gambia, Serra Leoa and Mexico.

Among the women who did not travel, two of them were collectors of natural history, for example Anna Jabonowska (1728–1800), who curated a very important European cabinet of natural curiosities. Her cabinet was considered one of the most important natural history collections in Europe in the eighteenth century [55]. The other ones (21%) worked in their countries, in particular Marie Le Masson Le Golft (1749–1826), whose work focused on marine biology; she published many books, including Coup d'Oeil Sur l'État Ancien et Présent du Havre (1778), with notes on fauna and flora of a French harbor [88, 89].

Ten of them traveled and/or published works with their husbands (35%). However, some of these women persisted traveling after their husbands’ death, such as Ottile Coudreau (1870–1910), who assumed control of an expedition in the Amazon region, publishing alone the results [92–96]. Eight of these female naturalists (25%) traveled by their own pursuits, such as Ida Pfeiffer (1795–1858) who traveled during 15 years around the world, coming to Brazil in 1846. She carried a letter of recommendation from Humboldt and other naturalists, helping her with her expeditions; she published many books that were translated to several languages [72]. Dorothea Maria Gsell (1678–1743), in particular, did not travel with her husband and neither traveled by her own pursuit, but traveled as an assistant of her mother, Maria Sibylla Merian. Later, she became professor of Arts in the Saint Petersburg’s Academy of Art and counselor of arts acquisition of Czar Peter. Also, she became administrator of Czar Peter’s natural history collection [62].

Six of them (21%) where traveler writers who made observations about plants in their publications, such as Baroness E. de Langsdorff (1812–1889), who came to Brazil in 1842 with the aim of dealing with the marriage of Prince of Joinville and Princess Francisca, Brazilian Emperor D. Pedro I’s sister; Langsdorff published her observations on landscapes and plants in a journal [56]. Teresa von Bayern, Princess of Bayern (1850–1925) [100] and Maria Graham (1785–1842) [80] collected plants that were included in *Flora Brasiliensis* of the German naturalist Carl Friedrich Philipp von Martius [101].

Most interestingly is to note that the trajectory of every one of these women is really admirable. Adela Breton (1849–1923), for example, was an archeologist, explorer and artist. She registered an archeological site in Mexico, including observations on landscape and vegetation. Her work is recognized as of great importance for Mesoamerican studies. Breton also made many watercolors of plants native to Central America, mainly Mexico [50]. Jeanne Barret (1740–1897) traveled disguised as a man, being the first woman ever to travel around the world in a scientific expedition. She was the botanist assistant of Philibert Commerson [77]. Marianne North (1830–1890) carried out many scientific expeditions aimed at illustrating plants in their natural environment, illustrating more than 700 plant genres. Some were totally unknown and were named after her, such as *Northia seychellana*, *Nepenthes northiana* and *Crinum nothianum*, the last one was described based on her drawings [82, 83].

However, something in common is the ethnobotanical data in their works, which still did not receive necessary attention.

### The case of Maria Sibylla Merian

#### Maria Sibylla’s erasure mechanisms

Maria Sibylla Merian's trajectory draws a lot of attention, although her scientific relevance has been neglected during the last centuries, mainly outside Europe. After all, she managed to carry out a self-financed scientific expedition in the seventeenth century, publish her work and achieve recognition by her contemporaries, being quoted by Linnaeus [[102], page 293] and Goethe [41, 63]. Her name is on the façade of the Artis Library at the University of Amsterdam along with 35 men of science [62]. She was one of the few women to be mentioned by the Royal Society at that time, having her work advertised in the first scientific journal, the Philosophical Transactions [103].

Maria Sibylla, alongside James Petiver,^2^ made several efforts to publish an English version of *Metamorphosis Insectorum Surinamensium* but was unable to do so. The limited number of publication languages is one of the reasons for this relative obscurity [104]. However, several editions were published in German, Dutch, Latin and French. Hans Sloane, later president of the Royal Society, acquired one of the first editions [105]. Thus, the language limitation does not seem to have been sufficient to suppress one of the first detailed texts in the field of Entomology.

In the eighteenth century, Maria Sibylla was widely admired for her contributions to art, natural science and exploration. However, in the nineteenth century, the major institutionalization of science contributed to the official exclusion of women from scientific practices [106]. Many women voyagers were concealed because they transgressed the expected image of a respectable woman at the time [38], including the Merian’s work [45]. In the twenty-first century, feminist movements and researchers have rescued Merian from oblivion [30, 48, 60, 62]. The question that remains about Maria Sibylla's trajectory is how and why, after reaching a level of scientific recognition in the seventeenth and eighteenth centuries, she is erased in the nineteenth century and until today she does not receive the same level of attention as other male naturalists?

In *How to suppress women’s writing* (1983), Joanna Russ [107] describes how women’s contributions to literature have suffered systematic devaluation and reports patterns in their suppression. In Maria Sibylla Merian's trajectory, which went from recognition to ignorance, it is possible to recognize these same mechanisms, with a similar pattern of erasure of women in literature and in science.

The first key area of suppression, described by Russ, is formal and informal prohibition. Merian only had access to natural history books and contact with botanists because she was the daughter of an important book publisher. Matthaus Merian, her father, published some of the most influential texts on natural history in the 1600 s, and through her brothers she also learned to engrave on copper plates, which allowed her to publish her own works and thus finance her expedition [45]. Had it not been for her stepfather Jacob Marrel, Merian would not have had access to artistic training. At the time, women could not become artists' apprentices [41]. After leaving the Labadist community, she moved to Amsterdam because it was one of the only places that allowed women to own a business [64]. In addition to formal bans, informal ones such as discouragement are also strong deterrents. It is possible to see in the preface of one of her first works that Merian was discouraged, saying that she did not feel safe making discoveries because she was only “a young woman” [44, 108].

After managing to overcome the barrier of prohibitions and to write her work, Russ [107] reports that the next tool for suppression is to deny authorship, stating that she did not write it, attributing it to someone else (usually a male). Even though her focus was on entomology and her illustrations revolved around her discussion of metamorphosis, Maria Sibylla Merian, for many years, was seen as an artist rather than the scientist she was. In a 1997 catalog of an exhibition of Merian's work [109], it is said that her work should be read as artistic and not scientific since she was not an academic and did not have the necessary qualifications to be the author of a scientific work: “Merian was not a scholar. She was a painter, an embroideress, a dealer in paints, a teacher, a housewife, and a mother and a lover of nature. She came from a family of craftsmen, had not studied at a university, knew little Latin and basically had none of the qualifications required of an author of a scientific work.”

**Fig. 4:**
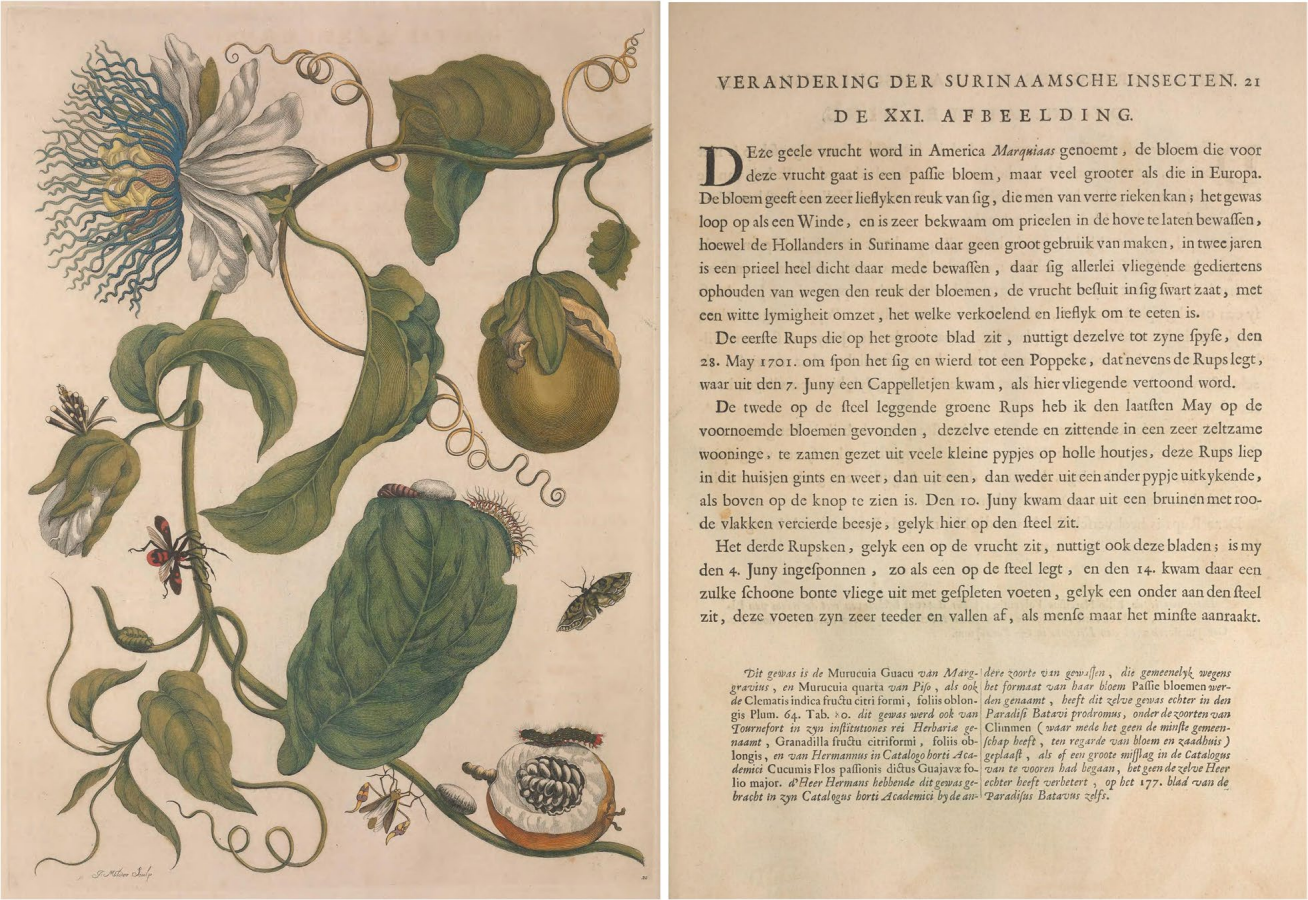
Illustration 21 (left) and description (right) about *Passiflora laurifolia* L. in Maria Sibylla Merian’s *Metamorphosis Insectorum Surinamensium* (1705) [7]

She was seen by many as a skilled artist, but when she published her work *Metamorphosis Insectorum Surinamensium* [7], she made it clear that she saw herself as a naturalist. Like many others, she had no formal academic training and studied in Amsterdam's collections and library and read and cited the work of Leeuwenhoek, Moffet, Swammerdam, Goedart and other distinguished scientists [110]. In addition to having devoted great care and effort to correlating her work with that of other naturalists, such as Willem Piso [111]. For each plant, she gave an indigenous, Dutch, and Latin name prior to Linnaeus's taxonomy [112]. For example, the illustration 21 refers to *Passiflora laurifolia* L. (Passifloraceae) and Merian reported its vernacular name as “marquiaas” and also “passiebloem”. She also annotated the names given by Marcgraf (“Murucuia Guacu”) and Piso (“Murucuia quarta”) (Fig. 4).

The ultimate test for claiming that Maria Sibylla Merian is a scientist is the fact that plant species can be identified from her illustrations. The number of identifications is extraordinarily high, proof of the accuracy of her work and concern for scientific rigor rather than just an aesthetic issue, Merian's accuracy even surpasses that of several later naturalists [63]. The ultimately botanical identification was made by the botanists Tinde van Andel, Hajo Gernaat, Auke Hielkema and Paul Maas published in the 2016 facsimile edition of *Metamorphosis Insectorum Surinamensium* [48].

After the publication of her diary by the Russian Academy of Science [113], historians began to examine her contribution as a naturalist. They found out that, at the age of 13, Merian began observing the metamorphosis of the silkworm, reporting its entire life cycle starting from the egg. When she published her book on European caterpillars [44], the spontaneous generation of insects was still well accepted by academics and she wrote in her preface: “all caterpillars are born from eggs”. She made discoveries independently and at the same time (sometimes even before) as other renowned scientists such as Redi, Malpighi and Swammerdam [104]. Her observations helped to discredit the spontaneous generation of insects [62].

**Fig. 5:**
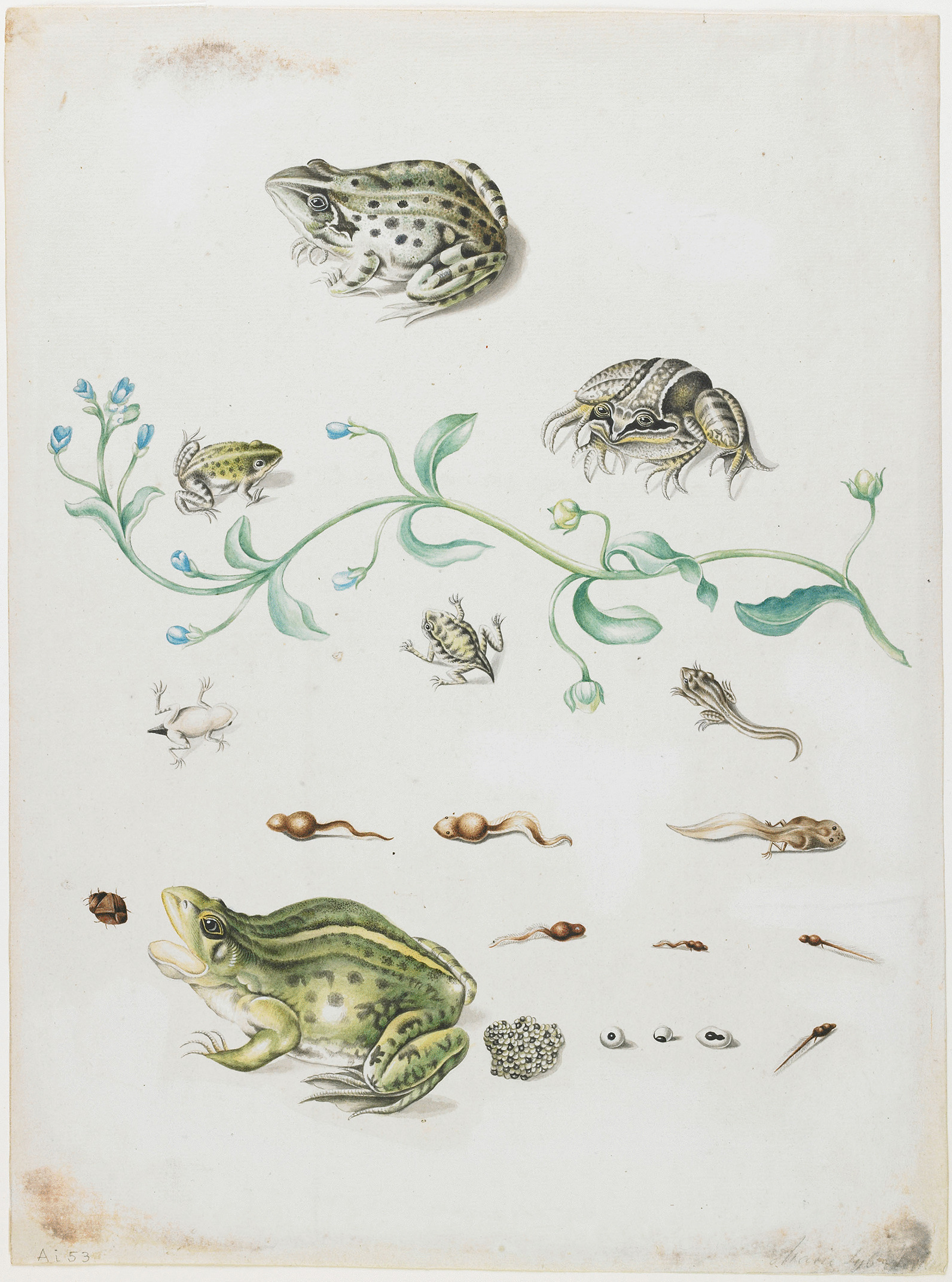
Metamorphosis of a frog and blue flower by Maria Sibylla Merian, circa 1701–1705. Watercolor on bodycolor on paper. The Minnich Collection The Ethel Morrison Van Derlip Fund. Minneapolis Institute of Arts (MIA) (https://collections.artsmia.org/art/10445/metamorphosis-of-a-frog-and-blue-flower-attributed-to-maria-sibylla-merian)

Merian was also one of the first naturalists to accurately describe the complete metamorphosis of amphibians [114] as is possible to see in Fig. 5. Her description is not as detailed as that by Leeuweenhoek and Swammerdam (who are credited), but she probably did not have access to a microscope like they did—again an informal ban aiding the obliteration of a female scientist [110]. She described the development of frog eggs and tadpole metamorphosis in her diary in 1686 [113], more than a decade before Leeuwenhoek observed the same phenomenon and reported it to the Royal Society on September 25, 1699 [115], and she provided the first representation of the amphibian *Pipa pipa* and *Trachycephalus venulosus* [110].

Another authorship denied to Merian is the observation and report of one of the first determinations of the mechanism of parasitism and parasitoidism [44]. She was the first to observe and document the metamorphosis of tropical insects, the first to illustrate many of the tropical plants in detail, and one of the first to offer a color edition of tropical species. For example, she first documented the plant *Inga edulis* [7, 48].

Linnaeus, considered to be the father of modern taxonomy, used Merian's work to name more than one hundred species, with Maria Sibylla's work being one of the main references for tropical species of insects and plants [10, 62, 104, 111, 114]. Over the centuries, species names have been substituted. The specific epithet *merianella* has been abandoned. For example, *Eulamprotes wilkella* (Linnaeus, 1758), which replaced *Tinea merianella*, and *Micropterix aureatella* (Scopoli, 1763), which replaced *Phalaena merianella* [116]. One of the names of the frog *Trachycephalus venulosus*, which was illustrated for the first time by her, was *Rana meriana*, which credited the discovery to Maria Sibylla Merian [110]. The specific epithet “*merianella*” is still used, however, in minor frequency than initially.

Her work was copied in the eighteenth century and influenced many later naturalists, such as Eleazar Albin, August Johann, Rosel von Rosenhof and Mark Catesby. Rosenhof, in his work on German frogs [117], included images of frogs in their habitat that resemble her work. Eleazar Albin referred to Maria Sibylla's early books in his compositions [118]. Mark Catesby’s work “Natural History of Carolina, Florida and the Bahama Islands” [119] is similar to Sibylla's work in design, length and composition, but he only cites her work to criticize errors; he pointed out that she misunderstood the depiction of the cashew nut. It is well established that all the naturalists cited were familiar with her work [110].

Natural history was becoming an academic discipline and, at the same time, the name of Maria Sibylla Merian was being suppressed from the history of science [63]. Unlike most naturalists of the seventeenth and eighteenth centuries, Merian acknowledged the help of enslave people, mainly African and indigenous women, in recognizing species and in gaining knowledge about the uses of plant species [[7], see the preface and illustration number 45]. Maria Sibylla's work is a compendium of women's knowledge written by a woman [111].

Etheridge [111] questions whether and how gender relations in Europe influenced knowledge about plants that naturalists collected in other cultures. In several communities, women and the elderly are essential in retaining traditional knowledge about medicinal plants [120]. This raises the possibility that inequality between men and women may have influenced the underestimation of the knowledge held and propagated by women.

Despite being related to different contexts and specific particularities, the way in which Maria Sibylla describes an abortifacient plant [7] stands out, differing in many aspects when compared to other male naturalists. Hans Sloane [11] and Alexander von Humboldt [121], for example, described abortifacient plants and it can be seen from their observations that, as a result of their time, these naturalists did not understand what could justify the use of this type of resource [60]. In contrast, Merian describes abortifacient plants as a resistance tool for enslaved women to control their reproduction [[7], see illustration number 45], transcribing a dialogue she had with women about believing that, by being aborted, their children would be born free [111].

This recognition of how unfair slavery was, present not only in the section about an abortifacient plant, also contributed to the erasure of the naturalist [60]. This position of hers was criticized, using another mechanism described by Russ [107], called pollution of agency, which consists of spreading the idea that women make themselves ridiculous by occupying spaces that were denied to them, showing them as neurotic, unpleasant, unlovable, abnormal, etc.

In the nineteenth century, Lansdown Guilding, a British naturalist best known for his work on Caribbean fauna and flora, published a review of Merian's work in the *Magazine of Natural History* (1834) [122]. His article was brutal and racist, openly criticizing her for crediting Africans and indigenous people who helped her. He called her work worthless, meaningless, unimportant, a judgment he justified based on his misconceptions. He cited errors in coloring, present only in non-original editions, such as a fruit that should be yellow (it was yellow in the original) and an insect that could not be red (it was not red), suggesting that she lied.

**Fig. 6:**
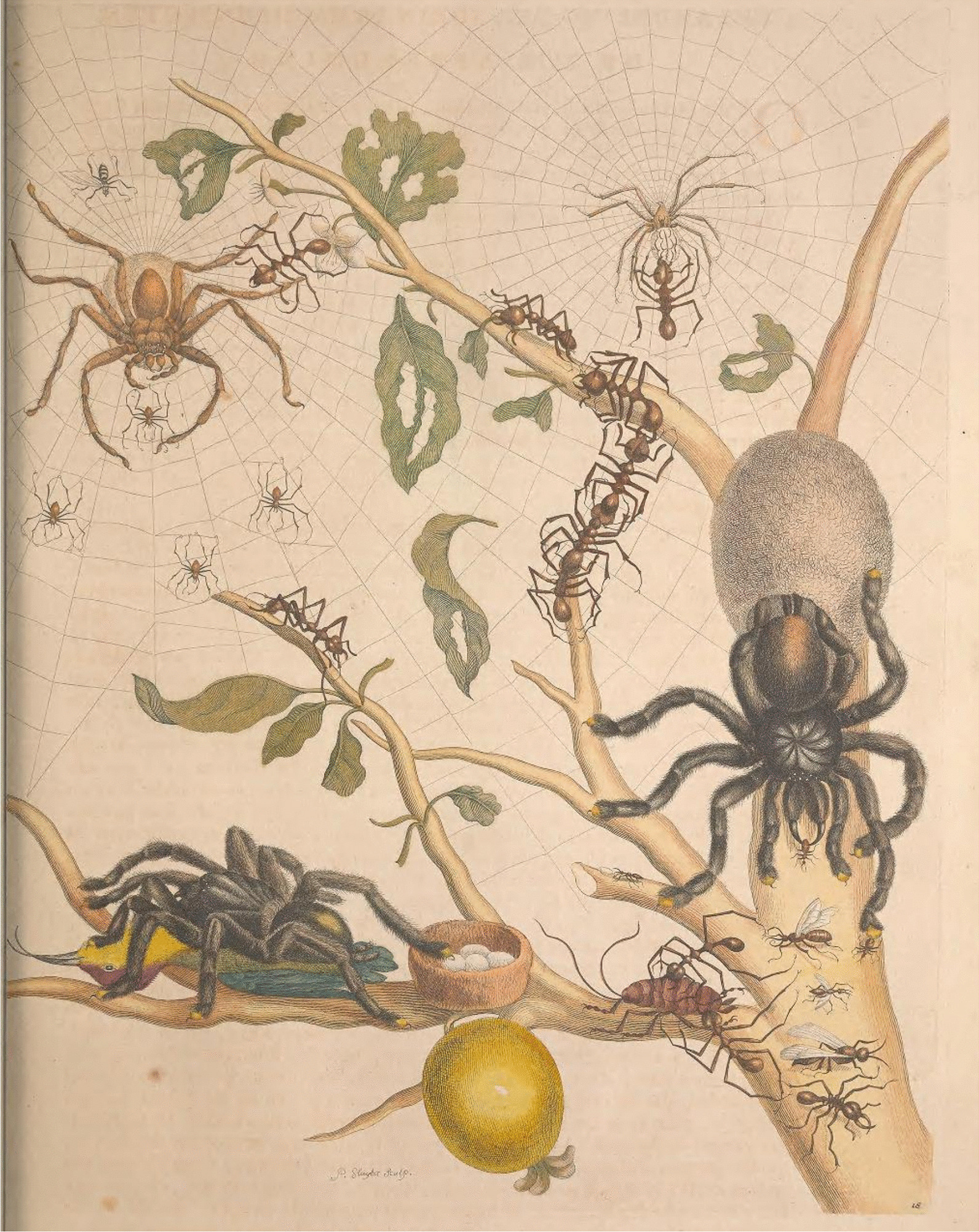
Illustration 18 showing a tarantula attacking a bird in Maria Sibylla Merian’s *Metamorphosis Insectorum Surinamensium* (1705) [7]

Contemporary reviewers criticized her for being a peculiar woman with strange interests, fearing that her travel to distant places would set a bad example for other women [114]. In an article published in 1834, William S. MacLeay, a British entomologist, criticized a book illustration for showing a tarantula attacking a bird (Fig. 6) [123]. Tests were conducted to prove that spiders could not attack a bird [63]. Currently, it is known that this is possible, including the description of spiders preying on birds in the Brazilian Amazon [124].

In William Jardine's *The Naturalist’s Library*, a collection of books on the biographies of important naturalists, the memoir of Maria Sibylla was written by James Duncan [[125], see pages 17–46], a Scottish naturalist who based all his work on the previously cited criticisms. Other naturalists became involved in the debate and destroyed Maria Sibylla Merian's work, although none of the critics ever went to South America [63].

In addition to criticism based on misconceptions and aimed at defaming her reputation, Merian's work has received a surprising amount of negative criticism directed at minor errors present in her work [114]. Errors are common and present in several more celebrated works [111]; however, looking for errors and highlighting them is also one of the tools described by Russ [107], called work depreciation.

**Fig. 7:**
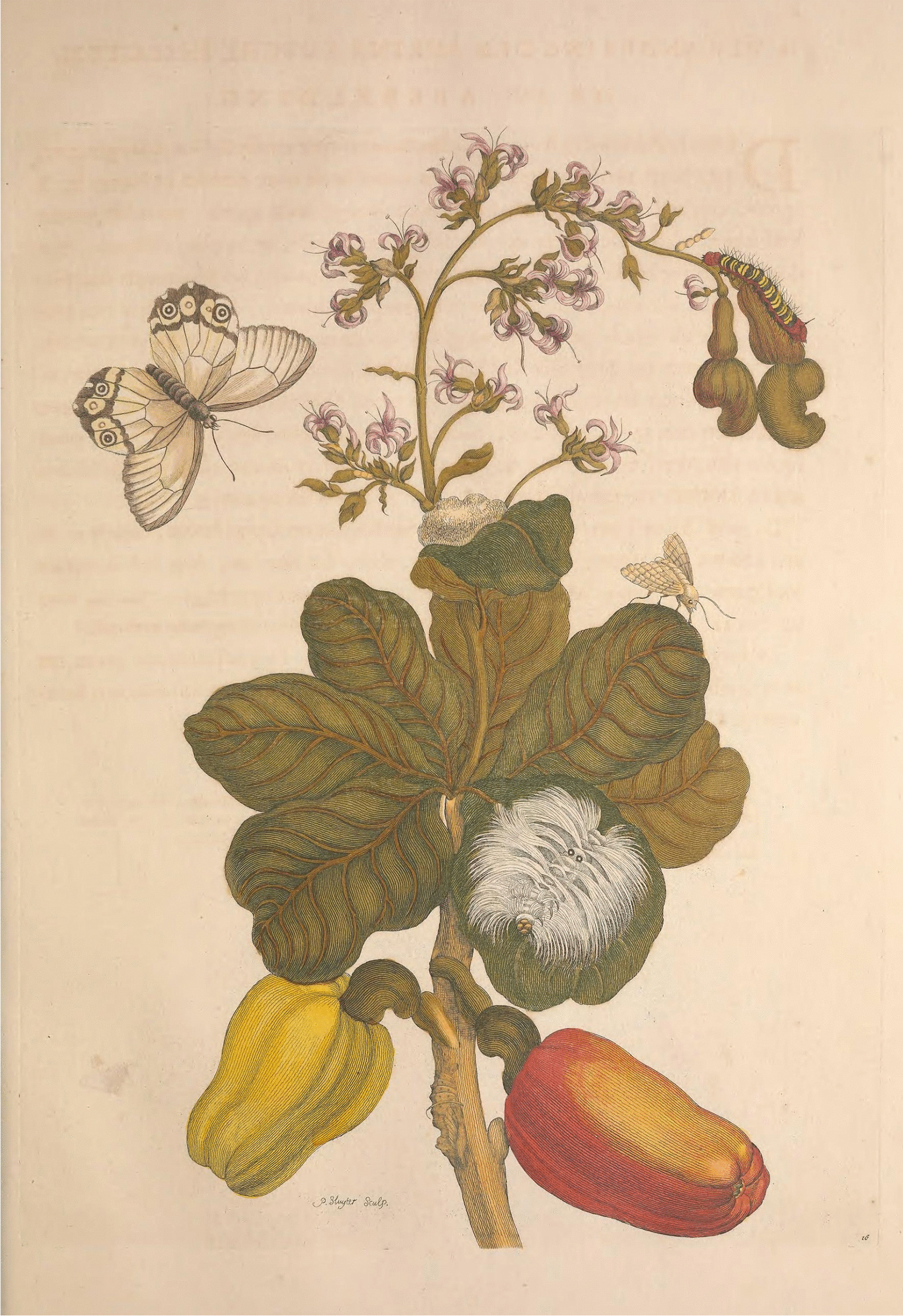
Illustration 16 representing cashew fruits (*Anacardium occidentale* L.) in Maria Sibylla Merian’s *Metamorphosis Insectorum Surinamensium* (1705) [7]

In fact, she made some mistakes in her illustrations, such as the position of the cashew in a representation in which the portrayed ripe cashews are connected to the branch by the nut and not by the peduncle (Fig. 7) [7], although the flowering branch, the unripe fruits and the ripe fruits themselves are well represented; probably she saw the ripe fruits on the ground and drew them. In another illustration, a butterfly larva species was connected with a moth species. These mistakes were probably unavoidable due to the difficult conditions under which she worked in Surinam and the relatively short time of her visit due to health reasons [114]. However, these small errors were used as a reason for the total depreciation of her work.

In a speech to the Royal Society, British naturalist George Edwards cited Merian's work, but specifically highlighted her description of the process of metamorphosis of an erroneously portrayed frog, and from that single error, he declared that she was unable to correctly observe the way nature acts [126]. In addition to the fact that a single illustration cannot belittle or discredit a work, this specific illustration was not even made by Maria Sibylla. When she died, Dorothea Merian (her youngest daughter) sold the rights to publisher Joannes Oosterwijk, who added 12 figures to the 1719 edition. Most appear to have been based on images drawn by Merian, but at least two were not her work, including the one criticized by Edwards. But as subsequent copies were printed more times than the original, this error was associated with her and damaged her reputation for accurate observation [110].

Another tool for suppressing women in literature, which can be seen as part of obliterating Maria Sibylla Merian's importance to science, is the content standard. Men and women, white and black people, LGBTQIAPN+ and heterosexual people, people from different social classes have access (or are denied access) to different opportunities, as well as facing different obstacles and discrimination [107], resulting in different life experiences and perspectives, which are socially reinforced. And it is to be expected that these differences will be reflected in the work that is produced by these individuals. The problem is labeling one experience more valuable and more important than others [107].

At a time when scientists were trying to make sense of nature by classifying plants and animals into separate categories, Merian looked at nature with a broad view looking for connections. She did not draw the specimens on an isolated and empty background, she portrayed animals as living organisms residing in a habitat, highlighting the relationships between animals and plants. She wanted to observe the metamorphosis of insects and their interaction with the habitat, presenting her objects of study as part of a food chain [110]. She was also the first to portray the life cycle of insects along with their host plants, emphasizing the interaction of the represented species, which is the foundation of ecology [104].

Her illustrations are spectacular and attract attention but, being different from the standard of the time, they were the target of much criticism and were reduced to artistic illustrations [114]. Paradoxically, the aesthetic quality of her work may have contributed to the idea that Merian was not a serious naturalist [104].

It is very difficult to find reports and analyses of other works by Maria Sibylla Merian, in addition to *Metamorphosis Insectorum Surinamensium*, although she published three editions of a book on European insects and other works [43, 44]. This is the result of another tool described by Russ [107], i.e., isolation. When an author's work manages to reach the level of relevance, this achievement is portrayed as isolated and the other works are considered non-existent or inferior. Over 50 years, she collected and studied caterpillars in a first unprecedented long-term zoological study that yielded several publications [63]. However, only the work she did in Surinam achieved some prestige.

Anomaly, another tool present in the suppression of women, consists of treating a certain woman as an exception of her gender [107]. This occurs in the scientific academy and a classic example is the Marie Curie effect, described by several historians in the twentieth century. The possibility has been raised that Marie Sklodowska Curie's celebrated achievements made it harder for women to enter science rather than opening doors. Because she was so brilliant, it was asked if this did not raise the bar of comparison too much, leading laboratory heads to only accept the next Marie Curie [127]. This effect ceases to make sense when we look at celebrated male scientists who did not make it difficult for other male scientists to be accepted [127]. However, to prevent Maria Sibylla Merian from being taken as an exception and a unique woman naturalist, one of the objectives of the present study was to carry out a survey of other women naturalists.

The last tool for the systematic devaluation of women in literature is the lack of role models. Models work as guides and indicate possibilities, and being inspired by women is very valuable. Facing continuing and massive discouragement, women need role models not only to see that it is possible to be an artist or a scientist as a woman, but to ensure that they can produce art and science without inevitably being devalued or socially excluded [107].

To ignore or erase the contribution of women to the history of science is to deny models for women scientists to look up to. A 28 year study of publications in the Brazilian ethnobotanical literature demonstrated that, although there is no difference between the number of female and male first authors, senior male authors publish more articles and in journals of greater impact compared to female senior authors [128]. That same study conducted interviews with Brazilian ethnobotanists and reported that 53.2% of the interviewees felt they were being discriminated in the academic environment for being women, with manterrupting (being constantly interrupted by a man without being able to present or complete an idea), being the most frequent event [128]. Pietri et al. [129] demonstrated that informing women about gender bias increases their identification as female scientists, protecting them from the consequences associated with this bias, such as reduced confidence, which makes it even more difficult for a woman scientist to impose her opinion after several interruptions.

This gender bias is the result of the historical persistence of male gender privilege in the role of scientist compared to female gender [128]. Calaza et al. [130] draw attention to the key role of implicit bias in the fight against gender discrimination. Bias is a concept that refers to analysis, judgment or attitudes that do not follow the principles of impartiality. Bias against a person or group of individuals can lead to an unfair evaluation, and can be implicit (when it is not noticed)—this being the most prevalent case-, or explicit.

As implicit bias is more prevalent, it is essential to raise awareness about what is often ignored [130], a very important attitude in order to rescue women scientists and talk about them. Mainly, this involves highlighting the gender bias present in the scientific academy regarding the way the history of science is told, in particular, from the perspective of naturalists who contributed to the knowledge of useful plants in South America.

Raising awareness of gender bias and increasing the representation of women scientists in research spaces and positions are effective ways to improve the current scenario, by countering gendered downplay mechanisms. These actions also provide opportunities to recognize women's contributions to knowledge production and to create models that can serve as guides and inspire future generations of female scientists. By promoting diversity and gender equity, we can encourage more women to pursue careers in science and provide tools to address gender bias [107, 128, 129].

### Useful plants in *Metamorphosis Insectorum Surinamensium*

**Fig. 8:**
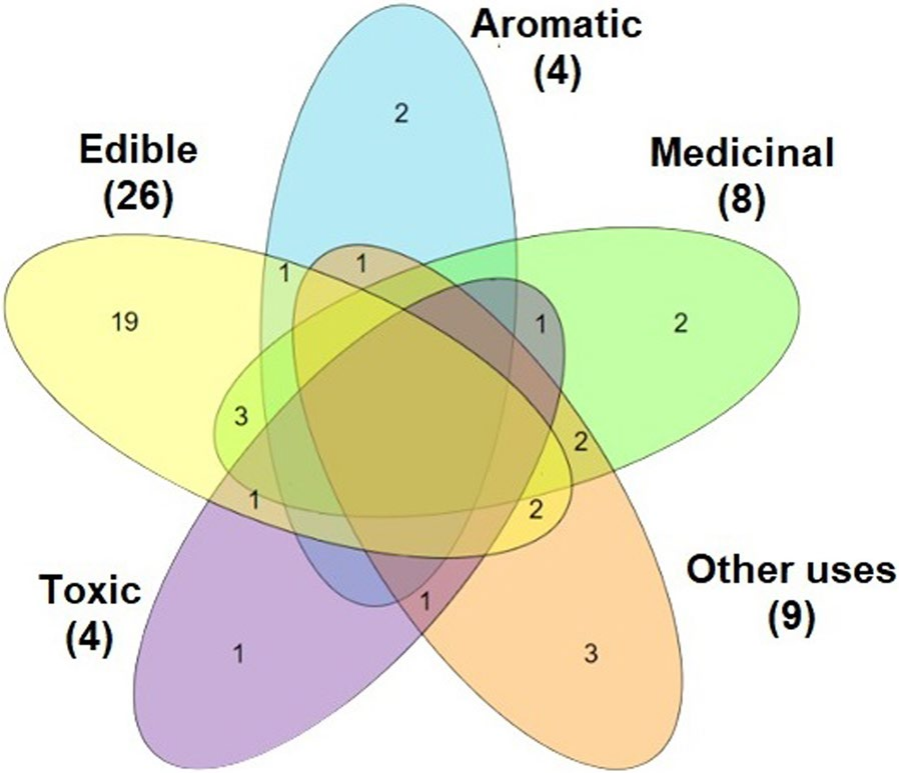
Venn’s Diagram illustrates plant categories related to traditional use registered in *Metamorphosis Insectorum Surinamensium* (1705) by Maria Sibylla Merian

*Metamorphosis Insectorum Surinamensium* contains 60 illustrations; all of them portraying plants belonging to 54 different species [7, 48]. Besides providing illustrations, Merian registered vernacular names (Dutch names as well indigenous and African names), names cited by other naturalists, general descriptions and traditional uses of the plants. Regarding traditional uses, 26 plants were categorized as edible/food, 8 as medicinal, 4 as aromatic, 4 as toxic, and 9 as otherwise useful (Fig. 8; Tables 2 and 3).

**Table 2:** Medicinal and toxic plants described in *Metamorphosis Insectorum Surinamensium* (1705) by Maria Sibylla Merian

Family and species	Illustration	Original vernacular name^a^	Part	Origin^b^	Traditional use	Pharmacological evidence^c^
ANACARDIACEAE *Anacardium occidentale* L.	16	Caschou Boom, Caschou Appels [cashew]	Fruit (nut)	South America and Caribbean	Good as a vermifuge	Antiparasitic activity [131–138]
CUCURBITACEAE *Citrullus lanatus* (Thunb.) Matsum. & Nakai	15	Water Meloenen [watermelon]	Fruit	North Africa and Oriental Africa	Useful for recovery from sickness	Source of vitamins and minerals [139]; antidiarrheal activity [140]; diuretic and antiurolithic activities [141]; anti-inflammatory activity [142]; increases recovery in cases of fatigue, malnutrition and indigestion [143]
EUPHORBIACEAE *Jatropha gossypiifolia* L.	38	Not mentioned [bellyache bush]	Leaf	South and Central America and Caribbean	Purgative; decoction of its leaves is used to treat Beljack lethargic disease (disentery)	Purgative [144–146]
*Manihot esculenta* Crantz	4; 5	Manihot, Manyot, Cassave, Cassava [cassava]	Root	South America	Juice contains a poison; if consumed, both animals and humans die in pain	Neurotoxicity caused by cyanogenic glycosides [147–152]
*Ricinus communis* L.	30	Palma Christi, Olyboom [castor oil plant]	Seed oil	East Africa	Seeds when boiled in water release an oil which is applied over any kind of wounds	Skin wound healing [153–155]; anti-inflammatory and analgesic activities [156]
FABACEAE *Senna obtusifolia* (L.) H.S.Irwin & Barneby	32	Slaapertjes [sicklepod]	Leaf	South and Central America, Caribbean and Barlavento Islands (West Africa)	Leaves are used to cover wounds, helping healing	Anti-inflammatory and wound and fracture healing activities [157–161]
*Caesalpinia pulcherrima* (L.) Sw	45	Flos Pavonis, [peacock flower]	Seed	Central America	Seeds are given to women to accelerate labor; enslaved black and indigenous women look for this plant for abortion	Sedative, emmenagogue, dysmenorrhea [162]; anti-inflammatory activity [163, 164]; antifertility effect [165–167]
MALVACEAE *Gossypium barbadense* L.	10	Surinaamse Cattoen Boom [Sea Island cotton, creole cotton]	Leaf	South America	Leaves are used for refreshment and wound healing	Analgesic activity [168, 169]; refreshment [170, 171]; wound and burn healing [172]
RUBIACEAE *Genipa americana* L.	48	Tabrouba [genip, genipap]	Fruit	South and Central America, Caribbean and Barlavento Islands (West Africa)	Indigenous people believe the fruit is poisonous	Not found
*Duroia eriopila* L.f	43	Marmelade-Doosies-Boom	Trunk	South America	Trunk protrusions are medicinal for lung diseases	Not found
SOLANACEAE *Solanum mammosum* L.	27	Appel van Sodom [nipplefruit]	Fruit	South and Central America, Caribbean and Barlavento Islands (West Africa)	Deadly poison for both animals and humans	Very toxic plant due to its steroidal glycoalkaloids [173]

a) Original vernacular names and English names according to *Metamorphosis Insectorum Surinamensium* facsimile edition (2016) [48]; b) origin of the plants searched in Plants of The World online; c) pharmacological evidence searched in Pubmed, Science Direct and Google Scholar databases

**Table 3:** Edible and aromatic plants as well as species with other uses described in *Metamorphosis Insectorum Surinamensium* (1705) by Maria Sibylla Merian

Family and species	Ilustration	Original vernacular name^a^	Part	Origin^b^	Traditional use
ANNONACEAE *Annona muricata* L.	14	Zuursak, Suursak [soursop]	Fruit	South and Central America, and Caribbean	Delicious taste both in natura and cooked; before removing the peel, a meal is prepared with water and sugar; on Barbados Island, wine is produced from this fruit
AIZOACEAE *Sesuvium portulacastrum* (L.) L	59	Water-Kersse [sea purslane]	Flowers; leaf	global tropical distribution	Succulent thick and soft leaves for salad
ANACARDIACEAE *Spondias mombin* L.	13	Amerikaanse Pruimboom [Java plum tree]	Fruit	South and Central America, Caribbean and Barlavento Islands (West Africa)	Fruit is first yellow and astringent, but then becomes sweet
*Anacardium occidentale* L.	16	Caschou Boom, Caschou Appels [cashew]	Fruit	South and Central America, Caribbean	Astringent and acid taste, good when cooked; the wine made from this fruit is good but strong, causing alcohol intoxication
APOCYNACEAE *Plumeria rubra* L.	8	Indiaansche Jasmynboom [frangipani]	Flower	South and Central America, Caribbean	Flowers have a pleasant aroma
BIXACEAE *Bixa orellana* L.	44	Rocu [anatto]	Seed	South and Central America, Caribbean	Indigenous people crush and macerate the seeds, which yield a red ink after mixing with water, with the red color remaining even after drying; the natives use the red ink as an excellent ornament to paint important figures on their naked bodies
BROMELIACEAE *Ananas comosus* (L.) Merr	1; 2	Ananas [pineapple]	Fruit	South and Central America, Caribbean	Sweet fruit considered by Merian as the king of all edible fruits; it is consumed both raw and cooked; the delicious juice is extracted by pressing and distillation
BURSERACEAE *Bursera simaruba* (L.) Sarg	20	Gummi Gutta Boomen [tourist tree, West Indian birch]	Resin	South and Central America, Caribbean and Barlavento Islands (West Africa)	Used by painters
CARICACEAE *Carica papaya* L.	40	Papay-boom [pawpaw, papaya]	Fruit; trunk	South and Central America, Caribbean	The fruit has a pleasant taste and melts in the mouth; the trunk is emptied and used as a pipe to transport rain water to cisterns on the roof of the houses
CONVOLVULACEAE *Ipomoea batatas* (L.) Lam	41, below	Battattes [sweet potato]	Root	Mexico	Boiled with meat
CUCURBITACEAE *Citrullus lanatus* (Thunb.) Matsum. & Nakai	15	Water Meloenen [watermelon]	Fruit	North and East Africa	Delicious taste; pulp is very glossy and melts in the mouth like sugar
EUPHORBIACEAE *Manihot esculenta* Crantz	4; 5	Manihot, Manyot, Cassave, Cassava [cassava]	Root	South America	Bread is made from the root; before making the bread, all the juice must be removed and, after boiling, it becomes an extraordinary beverage; a mashed root mixture can be placed on thin metal plates over low heat and made into a cake
*Ricinus communis* L.	30	Palma Christi, Olyboom [castor oil plant]	Seed oil	West Africa	Water-boiled seeds release an oil used in lamps that burn all night
FABACEAE *Erythrina fusca* Lour	11	Palissaden Boom [coral bean]	Stem; seed	global tropical distribution	The heads of seeds are used as a hair brush; planks are made from wood and used to build houses or huts
*Inga edulis* Mart	51	Zoete-boontjes, Wycke-bockjes	Seed	South and Central America, Caribbean and Barlavento Islands (West Africa)	A viscous substance around the seeds is very sweet
*Inga* sp.	58	Zoete Boonen-Boom	Seed	South America	Beans (seeds) are covered by a white substance with excellent sweet taste
GESNERIACEAE *Drymonia serrulata* (Jacq.) Mart	53	Mispel	Fruit	South, Central and North America	Edible substance, that has the shape of a heart, in the middle of the fruit
MALPIGHIACEAE *Malpighia glabra* L.	7	Amerikaansche Kerschen [Barbados cherry]	Fruit	South, Central and North America, Caribbean	No European fruit is comparable in taste; the fruit is better than cherry
MALVACEAE *Abelmoschus moschatus* Medik	42	Muscus Bloem [musk mallow]	Leaf; flowers	East, Southeast and Meridional Asia	Indigenous maidens make bracelets with the flowers, using them as great ornaments; they feed and fatten chickens with leaves; flowers have a strong musky aroma
*Gossypium barbadense* L.	10	Surinaamse Cattoen Boom [Sea Island cotton, creole cotton]	Leaf; fiber (cotton)	South America	With cotton, a thread is made and used to weave the hammocks where the indigenous people sleep
*Abelmoschus esculentus* (L.) Moench	37	Okkerum, Althea [okra]	Fruit	Central and West Africa	When fruits open they leak a milky liquid that is used to prepare beverages
MORACEAE *Ficus carica* L.	33	Vygen, Vygeboom [fig tree]	Fruit	South, Southwest, Central and Southeast Asia, Southern Europe	Pleasant tasting and refreshing fruit, very beneficial for the inhabitants of these warm countries
MUSACEAE *Musa* sp.	12; 23	Banana, Baccoves, Bannanes [banana]	Fruit; leaf	Southeast Asia	It tastes good both in natura and cooked; the leaves are used to carry the breads to the oven; it is also used to prepare vinegar with water and sugar
MYRTACEAE *Psidium guajava* L.	18; 19; 57	Guajaves, Guaiaves [guava]	Fruit	South and Central America, Caribbean and Barlavento Islands (West Africa)	The fruit tastes good both raw and cooked; when cooked, all seeds and juice are removed with a spoon and then used to make cheesecake and preserves
OLEACEAE *Jasminum grandiflorum* L.	46	Welriekende Jasmin [Spanish jasmine]	Flowers	East Africa, South, East and Southwest Asia	Very strong smell that can be perceived from long distances
ORCHIDACEAE *Vanilla planifolia* Andrews	25	Banille [vanilla]	Fruit	South and Central America, Caribbean	Sweet oil is used to prepare a beverage called succolata
PASSIFLORACEAE *Passiflora laurifolia* L.	21	Marquiaas, Passiebloem [passion fruit, yellow granadilla]	Fruit; flower	South and Central America, Caribbean and Barlavento Islands (West Africa), Southeast Asia	Strong aroma that can be perceived from long distances; very good and refreshing taste
RUBIACEAE *Duroia eriopila* L.f	43	Marmelade-Doosies-Boom	Trunk; fruit	South America	The inner part of the fruit is removed and eaten
*Genipa americana *L.	48	Tabrouba [genip, genipap]	Seed; fruit; trunk	South and Central America, Caribbean and Barlavento Islands (West Africa),	The stem is boiled to prepare a dish that tastes better than artichokes; indigenous people press the juice and leave it in the sun to change to a black color and they use it to paint their bodies with different figures; within a day, this dye cannot be washed with any soap; then, within a short time, the dye becomes entirely fixed as a permanent non-removable ornament; when the trunk is cut, it releases a milky liquid which is used by the indigenous people to anoint themselves
RUTACEAE *Citrus* × *aurantiifolia* (Christm.) Swingle	17	Limmetjens [lime]	Fruit	Artificial hybrid	The natives eat it with almost all sorts of meat; the fruit contains an oil that is called precious, but has no known utility
*Citrus medica* L.	28	Citroenen [citron]	Fruit	South and Southeast Asia	Fruits with a thicker peel taste worse
*Citrus maxima* (Burm.) Merr	29	Pompelmoes [pomelo, shaddock]	Fruit	Southeast and Southwest Asia	It is not so sweet as orange, but its pulp and peel are firmer; delicious taste
*Citrus sinensis* (L.) Osbeck	52	Appels van China-Boomen [orange]	Fruit	Artificial hybrid	Fruit is full of the most delicious juice
SOLANACEAE *Solanum stramoniifolium* Jacq	6	Maccai,	Fruit	East, Southeast and Southwest Asia	Fruit is consumed by both birds and men
*Capsicum annuum* L	55	Indiaanse Peper, Piement [hot pepper]	Fruit	Central America	For its strong and sharp taste, the natives rub it on bread before eating or season bread with its broth; the Dutch cut and eat it with meat or fish
VITACEAE *Vitis vinifera* L.	34; 47	Wyn-druiven, Witte Wyn-Druyven [grapevine]	Fruit	Europe, Southeast and Southwest Asia	Wine is made from the fruit

a) Original vernacular names and English names according to *Metamorphosis Insectorum Surinamensium* facsimile edition (2016) [48]; b) origin of the plants searched in Plants of The World online; c) pharmacological evidence searched in Pubmed, Science Direct and Google Scholar databases

The families with the greatest number of species citations are Fabaceae (6), Malvaceae (5), Rutaceae (4), Euphorbiaceae (3), Solanaceae (3), Anacardiaceae (2), Rubiaceae (2), Annonaceae (2), and Heliconiaceae (2). Most edible plant families cited are Rutaceae (4), Solanaceae, Fabaceae, Rubiaceae, and Anacardiaceae, each containing 2 species.

Besides useful plants, the following species are represented in her illustrations, although without any information regarding some kind of use: *Pachystachys coccinea* (Aubl.) Nees (Acanthaceae); *Hippeastrum puniceum* (Lam.) Voss (Amaryllidaceae); *Annona sericea* Dunal (Annonaceae); *Ipomoea alba* L. (Convolvulaceae); *Costus arabicus* L. (Costaceae); *Muellera monilis* (L.) M.J.Silva & A.M.G.Azevedo (Fabaceae); *Heliconia psittacorum* L.f. (Heliconiaceae); *Heliconia acuminata* A.Rich (Heliconiaceae); *Theobroma cacao* L. (Malvaceae); *Hibiscus mutabilis* L. (Malvaceae); *Ludwigia octovalvis* (Jacq.) P.H.Raven (Onagraceae); *Argemone mexicana* L. (Papaveraceae); *Pontederia crassipes* Mart. (Pontederiaceae) and *Punica granatum* L. (Lythraceae).

Maria Sibylla Merian described mainly edible plants, reporting in detail different tastes she tried in Surinam. She observed and registered daily habits and food customs, reporting about the fruits used to prepare wine, juices and beverages; those which should be eaten cooked and their preparation; those used in recipes like jams and salads; and the combinations of plants and spices used. Useful plants were also reported, such as those used to weave hammocks, for construction, as pipes to carry water to the cisterns, as a hairbrush, as paint for rituals, and even as ornaments.

Merian carefully observed the plants and their uses, criticizing the fact that the settlers only cared about sugarcane and that their lack of curiosity wasted the potential of the fertile land of Surinam where several plants already known could be cultivated and several others could be discovered. She reported everything with a critical view, including commenting that if there was interest and studies were carried out, it would not be necessary to import wine to Surinam; on the contrary, the local people could plant grapes which grow very well and within a short time, and they could even export wine to the Netherlands [7].

Although there is a rich source of cultural information about useful plants, mainly those that are edible, the discussion will be focused on medicinal and toxic species, based on the reported traditional uses described by Merian, while searching for correlated pharmacological evidence.

Merian reported that the oil of *Ricinus communis* L. seeds is applied to wounds. In Suriname the plant is still used for the same purpose [48]. An ethnopharmacological survey carried out in Tharu—one of the largest communities in Uttarakhand, India—pointed out that this species is the medicinal plant most used by the community to treat skin diseases [155]. In Tharu, a paste is made from the leaves of this plant, which is applied to wounds and seed oil is used to cure eczema. Another ethnobotanical study carried out in Kumaun Himalaya, also located in Uttarakhand, India, reported that the leaves are used to aid wound healing [153]. In southwestern Nigeria, the leaves are also made into a paste and used for wound healing. An in vivo study performed on rabbits observed that an extract of *R. communis* leaves reduced the healing time of skin wounds [153]. In addition, in vivo studies in mice observed that an extract of *R. communis* leaves has anti-inflammatory and analgesic activity [156].

*Jatropha gossypiifolia* L. is described by Maria Sibylla Merian as a remedy to treat the lethargic disease Beljack, a condition known nowadays as dysentery [174], in agreement with the vernacular name, bellyache bush. The oil present in the seeds of the genus is known for its purgative action and it has been reported that the leaves of some species, including *J. gossypiifolia,* have the same effect [146]. Several ethnobotanical studies have also cited this purgative effect [144] and an in vivo study on albino rats observed that the leaf extract has antipyretic and laxative activity [145].

Merian reported that the juice of cassava, *Manihot esculenta* Crantz, is a deadly poison. It is known that this plant has great nutritional importance; however, cyanogenic glycosides are found in high percentage in its roots and leaves, such as linamarin and lotaustralin. These active constituents have neurotoxic and neurological effects because, after undergoing hydrolysis, they release cyanide derivatives [151]. Cyanogenic compounds need to be removed by peeling, boiling, fermenting, and cooking the plant, with a loss of up to 70% of these toxic substances [151]. After this process, the plant is used to obtain flour for cakes and breads, as reported by Maria Sibylla Merian. An in vivo study on Wistar rats showed that daily consumption of cassava juice led to impaired motor skills and neurological damage in the same manner as observed in the group that received linamarin [152]. Another in vivo study using the same animal models observed that intra-hippocampal or intraperitoneal administrations of acetone cyanohydrin (linamarin metabolite) resulted in loss of locomotor capacity and impairment of renal and hepatic functions [149, 150]. The consumption of this plant has been associated with the development of neurological disorders, mainly related to motor and cognitive impairment. An epidemiological study demonstrated a relationship between Konzo, a neurodegenerative disease, and the chronic consumption of insufficiently processed *M. esculenta*, and biochemical and toxicological studies have suggested intoxication with cyanogenic glycosides [147]. The consumption of non-processed cassava has also been associated with the prevalence of tropical ataxic neuropathy, a disabling disease that also increases mortality [148].

An excerpt in which Maria Sibylla Merian describes the traditional use of *Caesalpinia pulcherrima* (L.) Sw. is very sensitive and deals with a very difficult subject: “The seeds are given to women in labor, because they speed up the birth. For this reason, the natives, who are harshly enslaved by the Dutch, look for this plant in order to abort, so that their children will not be born as slaves and will not have to endure the same suffering as their mothers. Black slaves brought from Angola and Guinea also use this plant for abortion. Sometimes they commit suicide because they believe that they will return free to their country and with their friends, as they have informed me” [7]. In a review of medicinal plants of ethnopharmacological importance from the country of Mauritius (East Africa) [167], the trunk, bark and flowers of *C. pulcherrima* are reported to be abortifacient. The stem and bark are also reported to have an emmenagogue effect. An in vivo study of female albino mice [166] showed the anti-implantation activity of the ethanolic extract of the leaves of this plant, supporting its traditional use as an antifertility agent. This in vivo antifertility effect was also observed with the use of the alcoholic extract of the bark in Wistar rats [175]. Aqueous, ether and chloroform extracts from the aerial parts of the plant also had an antifertility effect in female albino rats [165]. No correlated evidence was found for its use to accelerate childbirth, but in folk medicine it is considered a sedative and emmenagogue and is also used for the treatment of menstrual cramps [162].

Merian also reported the wound healing activity of *Senna obtusifolia* (L.) H.S.Irwin & Barneby, another species of the Fabaceae family. In vitro and in vivo studies showed the anti-inflammatory and/or wound healing activities of transgenic hairy roots [158] and aerial parts [157] of *S. obtusifolia*. An in vivo study with mice revealed that oral ingestion of the powder of the leaves of other species belonging to the same genus, *S. occidentalis* (L.) Link, has anti-inflammatory action, helping to reduce edema [159]. *S. occidentalis* also appears in an ethnopharmacological survey of plants used for wound healing in Dogonland, Mali (West Africa). In that study, traditional healers from over 20 villages were interviewed and reported that the decoction of its leaves is used to wash any type of wound and that the powder of ground leaves is applied to aid healing [160].

Cashew nut (*Anacardium occidentale* L.) is described by Merian as a vermifuge. Components extracted from the nut, such as anacardic acid, have an anti-*Trypanosoma cruzi* effect [136, 137], as well actions against echinococcosis [138, 176] and a larvicidal and antimicrobial effect [177]. The ethanolic extract of the bark of this plant showed an in vitro anthelmintic effect against the parasite *Onchocerca ochengi* [176]. In an endemic region of tegumentary leishmaniasis in Bahia State, Northeastern Brazil, *A. occidentale* was reported as one of the plants used for the treatment of leishmaniasis by most of the interviewees [134]. This plant is also used by some indigenous communities in the Amazon to treat leishmaniasis and the extract of the leaves and stem has been observed to have an anti-promastigote effect [131]. The ethanol extract of cashew nuts showed an in vitro effect against adult larvae of *Schistosoma mansoni* [132] and also had an in vitro anthelmintic effect against *Haemonchus contortus* larvae [133]. Also, intake of cashew nuts decreased *Haemonchus contortus* infection in sheep [135].

In the work of Maria Sibylla Merian, the traditional medicinal use of watermelon, *Citrullus lanatus* (Thunb.) Matsum. & Nakai, is described in a very generic way only as useful in the recovery of the sick. This reported improvement in recovery may be due to the fact that this plant is a great source of vitamins such as thiamine, riboflavin, folate and niacin, and of minerals such as potassium, magnesium, calcium and phosphorus. Watermelon consumption has several health benefits such as reduced heart disease, better control of high blood pressure, lower LDL and a cardioprotective effect [139]. Watermelon seeds contain several amino acids, vitamins and components with a potential protective effect on the lungs, liver and pancreas, in addition to having an antioxidant effect [178]. The seeds and pulp of *C. lanatus* are used in traditional medicine to treat gastrointestinal, respiratory and urinary diseases in India and Pakistan. In silico, in vitro and in vivo models have shown that the seed extract of this plant has an antiperistaltic and antidiarrheal effect, which is consistent with its traditional use [140]. These effects can be helpful in recovery from gastrointestinal illnesses. The pulp extract of this plant showed diuretic and antiurolytic activity in both in vitro and in vivo models. The pulp extract also prevented weight loss in mice with induced kidney stone formation compared to the group that received no treatment [141]. Weight maintenance is an important factor in recovery from illness. And the effect of diuretic and antiurolytic activity could justify the better recovery in some urinary diseases. In Sudan, *C. lanatus* is used in traditional medicine to treat rheumatism, swelling, gout and as a laxative. Cucurbitacin E, a bioactive compound isolated from the pulp of this plant, showed anti-inflammatory potential both in vitro and in vivo [142]. In traditional medicine, it is also recommended in cases of fatigue, malnutrition and indigestion, and is reported to improve recovery [143].

An ethnobotanical study carried out in Vale da Jurema (Mato Grosso, Brazil) described that fresh leaves of *Gossypium barbadense* L. are used in preparations for topical application to relieve pain [168]. Another ethnobotanical study carried out in Surinam reports the use of the leaves of this plant for perinatal health, refreshing and maintaining the health of the newborn's skin [171]. It has also been reported that in the Peruvian Amazon, the leaves are used to refresh and help with postpartum pain [170]. Preparations with the fresh leaves are also used for muscle pain and headache by the population of the Vale da Ribeira region (São Paulo, Brazil) [169]. This plant is also used to treat burns and wounds, as reported in an ethnopharmacological study carried out with plants for sale in popular markets in northeastern Brazil [172]. These data corroborated Merian’s descriptions of the wound healing and refreshing properties of *G. barbadense* leaves.

*Solanum mammosum* L. is a very toxic plant due to its composition in steroidal glycoalkaloids (e.g. solamargine) [173]. The yellow fruits of this shrub were sometimes the first fresh fruits that the hungry sailors saw in the seventeenth and eighteenth centuries when they finally arrived in Surinam after the long sea journey. They thought they were oranges, with which they could cure their scurvy and ate it greedily, leading them to death after excruciating pain. Nowadays it is used in Surinam against skin parasites [179].

*Genipa americana* L. and *Duroia eriopila* L.f. do not have any updated correlated information regarding their medicinal or toxic properties. Thus, it can be seen that there is evidence supporting the traditional use of 7 of the 8 medicinal plants and 3 of the 4 toxic plants reported by Maria Sibylla in her work, highlighting the importance of this historical record.

## Conclusion

In the present study we surveyed 28 women naturalists who contributed to the knowledge about useful plants. This study evidences and highlights that there are female naturalists whose works could provide valuable insights for ethnopharmacological studies. By analyzing the trajectory of Maria Sibylla Merian, we were able to highlight some of the mechanisms by which women scientists have been systematically excluded from the annals of science history. Researching and discussing the contributions of women scientists, as well as exposing the gender bias inherent to scientific academy in the way science history is narrated, are crucial steps toward building a more diverse and inclusive scientific community.

The work *Metamorphosis Insectorum Surinamensium* (1705) by Maria Sibylla Merian was analyzed in order to exemplify how much information is being lost by neglecting women naturalists. 54 plant species were listed and classified into edible, medicinal, aromatic, toxic and other uses plants. In the scientific literature, it was possible to find pharmacological studies correlated with the traditional uses of the medicinal and toxic plants reported by the naturalist. However, most of these studies are ethnopharmacological or in vitro/in vivo, emphasizing the need for clinical studies of these medicinal and toxic plants.

Highlighting and studying historical records of the use of medicinal and other uses plants provided by women naturalists such as Maria Sibylla Merian is important in order to direct strategic research about traditional medicine, promoting support for pharmacological research such as safety and effectiveness studies, in addition to reflections about conservation and environmental aspects.

## Data Availability

All data generated or analyzed during this study are included in this published article.

## Footnotes

1 This edition is one of the only translations of Maria Sibylla Merian's work into English, being an unofficial free translation into English handwritten in the eighteenth century by an unknown naturalist.

2 James Petiver was an entomologist, botanist, natural history collector, correspondent for several naturalists such Merian at the time, and a member of the Royal Society [105].
